# Formulation and Characterization of Carbopol-934 Based Kojic Acid-Loaded Smart Nanocrystals: A Solubility Enhancement Approach

**DOI:** 10.3390/polym14071489

**Published:** 2022-04-06

**Authors:** Barkat Ali Khan, Maryam Waheed, Khaled M. Hosny, Waleed Y. Rizg, Samar S. Murshid, Majed Alharbi, Muhammad Khalid Khan

**Affiliations:** 1 Drug Delivery and Cosmetic Lab (DDCL), Gomal Centre of Pharmaceutical Sciences, Faculty of Pharmacy, Gomal University, Dera Ismail Khan 29050, Pakistan; barkat.khan@gu.edu.pk (B.A.K.); shiekhmaryamwaheed@gmail.com (M.W.); khalid.gomalian@gmail.com (M.K.K.); 2 Department of Pharmaceutics, Faculty of Pharmacy, King Abdulaziz University, Jeddah 21589, Saudi Arabia; wrizq@kau.edu.sa; 3 Center of Excellence for Drug Research and Pharmaceutical Industries, King Abdulaziz University, Jeddah 21589, Saudi Arabia; 4 Department of Natural Products and Alternative Medicine, Faculty of Pharmacy, King Abdulaziz University, Jeddah 21589, Saudi Arabia; samurshid@kau.edu.sa; 5 Department of Pharmaceutical Chemistry, Faculty of Pharmacy, King Abdulaziz University, Jeddah 21589, Saudi Arabia; maaalharbi1@kau.edu.sa

**Keywords:** sustainability of natural resources, nanocrystals, kojic acid, solubility enhancement, top-down technique

## Abstract

Kojic acid (KA) is a BCS class II drug having low solubility and high permeability. This study was designed to enhance the aqueous solubility of KA, as well as its dissolution rate and, in turn, bioavailability, by formulating its smart nanocrystals. Nanocrystals of pure KA were formulated by the top-down method under high-pressure homogenization followed by freeze drying. The nanocrystals were evaluated for stability and other physical characteristics, including zeta sizer analysis, DSC, surface morphology, XRD, drug content, solubility, FTIR and in vitro drug release. The KA nanocrystals were found to be stable when kept at exaggerated conditions. The particle size of the nanocrystals was 137.5 ± 1.7, 150 ± 2.8, and 110 ± 3.0 nm for the F1, F2 and F3 formulations, respectively. There was negative zeta potential for all the formulations. The dispersity index was 0.45 ± 0.2, 0.36 ± 0.4 and 0.41 ± 1.5 for the F1, F2 and F3, respectively. The DSC studies showed that there was no interaction between the KA and the excipients of the nanocrystals. The morphological studies confirmed the presence of rough crystalline surfaces on the nanosized particles. XRD studies showed the successful preparation of nanocrystals. The drug content was in the official range of 90 ± 10%. The solubility of KA was significantly (*p* < 0.05) enhanced in the formulations of its nanocrystals as compared with pure KA powder. The ATR-FTIR studies revealed the presence of functional groups in both KA and KA-loaded nanocrystals, and no interaction was found between them. The nanocrystals released 83.93 ± 1.22% of KA in 24 h. The study concluded that the nanocrystals were successfully formulated using the top-down method followed by high-pressure homogenization. The solubility, as well as the dissolution, of the KA was enhanced, and this could improve the therapeutic effects of KA.

## 1. Introduction

At the present time, poor solubility is the leading limitation of drugs under development. Many modern chemical substances have good therapeutic effects and efficiency, but their clinical applications are constrained by their poor solubility. In the Biopharmaceutical Classification System (BCS), these novel organic substances belong to class II or class IV. More and more drugs are being found with issues related to solubility, as are new chemical entities with poor solubility. In total, 40% of drugs in the line and 70% of drugs in production have problems of solubility, and these subsequently lead to poor oral bioavailability and transport problems. Weak solubility results in unpredictable bioavailability and, thus, undesirable side effects [1]. Poor bioavailability correlates with poor solubility. If water solubility is not increased in any way, the drug will not be absorbed in the gastrointestinal system, get into the circulatory system and target the action site [2]. Different drug delivery systems are currently being used, yet there are still definite challenges that must be addressed, and innovative technology must be established for getting drugs to specific target sites [3]. Nanomedicine is currently considered an advanced drug delivery system that could achieve these goals [4].

Various physical and chemical methods are being adopted by pharmaceutical technologists for improving aqueous solubility and dissolution rates, which in turn could improve drug bioavailability. This involves the modification of drug characteristics at the particulate level [5]. By using physical strategies such as reducing the size of drug particles and by preparing drugs in a polymorphic form, colloidal drug dispersions and chemical techniques for salt formation, and soluble prodrugs with aqueous solubility, the poor solubility of some drugs can be improved [6].

Nanocrystals offer opportunities for linking nano-formulations with solid ingredients, including those with (1) a high load of active ingredients, (2) higher bioavailability and (3) lower systemic cytotoxicity [7]. Rapid dissolution is necessary for the rapid onset of drug action, and the oral administration of nanocrystals can accelerate the absorption and bioavailability of drugs [4]. The influence of food on drug absorption is also eliminated by the improved solubility of drug nanocrystals [8].

In order to maintain, sustainability of natural resources, Kojic acid (KA) can be used to protect against ultraviolet exposure and limit the effect of tyrosinase in causing excessive pigmentation. Skin whitening is the process of reducing the concentration of melanin in the skin, and, thus, hyperpigmentation, by the use of chemicals. Today, skin lightening is one of the most common ways of improving skin pigmentation [9]. KA and its derivatives are used to prevent melanocytes from forming pigment; KA is one of the most popular whitening agents in the cosmetic industry. Hydroquinone, a common whitening agent, has many negative effects, making it less suitable than KA [10]. KA is a known anti-tyrosinase agent that is effectively used to whiten skin and reduce hyperpigmentation. It is used as an ion chelator for a transitional metal (e.g., Cu^2+^ and Fe^3+^). It can also be used to treat wrinkles due to its ability to scavenge free radicals [11,12]. 

KA is a BCS class II drug with low water solubility and high permeability. Its poor solubility in water leads to a low dissolution rate, which leads to a lower concentration of the drug in the plasma and a sub-therapeutic response. The new nanocrystal technology is necessary to overcome deficiencies in hydrophilicity, solubility and dissolution rate.

With hopes of improving the aqueous solubility of KA, this study was designed to fabricate and characterize smart nanocrystals of KA.

## 2. Materials and Methods

### 2.1. Materials

KA was purchased from Shaanxi Sangherb Biotech (Shaanxi, China). The water-soluble polymer Carbopol-934 (poly acrylic acid with molecular weight average 450,000) and Tween-80 were supplied by Sigma Aldrich (Darmstadt, Germany). Distilled water was acquired from the research laboratory of Gomal University, D. I. K. (Khyber Pakhtoonkhwa, Pakistan), as were ethanol, acetone, phosphate buffer (pH 6.8 and 7.4) and methanol.

### 2.2. Fabrication of KA Nano-Suspension

KA nano-suspension was prepared by using Tween-80 followed by high-speed homogenization with the top-down method [13]. Carbopol-934 was dispersed in distilled water slowly to form solution A, which was mixed well and placed on a magnetic stirrer. Tween-80 was heated at 35 °C and dispersed in water for 30 min to form solution B. Solution C was formed by combining solution A and solution B for at least 1 h on a magnetic stirrer. After 30 min, KA was added to solution C. This solution was homogenized at high speed by the ULTRA-TURRAX D7813 (Staufen, Germany), that is, 15,000 revolutions for 15 min. KA nano-suspension was readily developed and stored in a refrigerator at 2 to 8 °C. The final composition of nanosuspension has been depicted in Table 1.

### 2.3. Stability Studies of Nano-Suspensions

To select stable KA nano-suspension and remove unstable, thermodynamic stability tests were carried out for blank and drug-loaded nano-suspension. The optimum temperatures at which any physical change could be observed were 40 °C and 4 °C over 24 h. Visual inspection of all aspects of the formulations’ instability was checked. The designated formulation was centrifuged with the SCILOGEX (BERLIN, NH, USA) at 4000 rpm for 15 min. The stable nano-suspensions were then subjected to the lyophilization process [13].

### 2.4. Lyophilization of Nano-Suspensions

Lyophilization is the process of converting a liquid into a solid form in a vacuum and at low temperature. A vacuum freeze dryer (BIOBASE, FD 10 S, Jinan, China) was used for the lyophilization process. The stable formulations (F1, F2 and F3) were subjected to the lyophilization process, converted into the powdered (Nanocrystals) forms and further evaluated for in vitro characterization [14].

### 2.5. Characterization of Nanocrystals

The optimized formulation was subjected to variouse physicochemical characterizaion like Particle Size, Zeta Potential, Dispersity Index (DI), DSC, XRD, SEM, content determination and solobility studies

### 2.6. Particle Size, Zeta Potential and Dispersity Index (DI) of Nanocrystals

Dynamic light scattering with the Zetasizer Nano ZS (Malvern Instruments, Malvern, WR14 1XZ. UK) laser Doppler velocimeter and a 90-degree detection angle at 25 °C was used for the characterization of the average particle size (PS), dispersity index (DI) and zeta potential (ZP). Each sample was analyzed in 14 runs, and the final result was reported as the average value plus the standard deviation (SD). The room temperature was kept fixed [10].

### 2.7. Differential Scanning Calorimetry Studies

To analyze the matrix structure and thermal behavior of KA-loaded nanocrystals, DSC with the Netzsh-200 PC (Burladingen, Germany) was used. Lyophilized samples of ATR-FTIR were considered. Approximately 5 mg of KA and lyophilized KA nanocrystals were placed on an aluminum tray. The sample was then sealed in a container, and an empty container was used as a control. DSC scanned the sample at 20 to 300 °C with a heating rate of 20 °C/min under N_2_ flow. The obtained DSC thermogram results were used to evaluate the interaction between the drug and the excipient. The melting point and enthalpy of the pure drug and the KA nanocrystals were also determined [14].

### 2.8. Scanning Electron Microscopy

A field emission scanning electron microscope (FESEMS3400N Hitachi, Tokyo, Japan) was used to examine the surface morphology of the KA nanocrystals. A sample of freeze-dried KA nanocrystals (0.5 g) was dissolved in 10 mL of distilled water. Then, 2 mL of the prepared nanocrystals was placed on a glass slide. The sample was placed in a desiccator to dry. Once it was completely dry, the sample was plated with gold to reduce the static charge during the examination [15].

### 2.9. X-ray Diffraction Studies

Powder X-ray diffraction (PXRD) was performed on the prepared KA nanocrystals to characterize the changes in the components of the lattice structure. The results of the DSC were confirmed by the XRD results. PXRD analysis was carried out with a Bruker D8 Advance (Berlin, Germany) X-ray diffractometer at 40 kV and 30 mA. The obtained PXRD pattern further confirmed the encapsulation of KA in the nanocrystals. The sample was exposed to Cu Km radiation (λ.0.15406 nm), and the apparatus was fitted with a sample rotator and operated at a current of 30 mA and a voltage of 40 kV. The measurement was carried out at room temperature and ranged from 1 degree to 100 inches at 20 degrees with a step length of 0.04 inches and a step length of 1s [15].

### 2.10. Drug Content of Nanocrystals

To determine the drug content, ethanol was used as a solvent. In total, 100 mg of KA nanocrystals was placed in a 100 mL volumetric flask. Ethanol was added, and the solution was vigorously stirred and allowed to stand at room temperature for 24 h with intermittent shaking. By centrifugation, the supernatant was collected. With UV spectrophotometry, the drug content was determined at a suitable wavelength (268 nm) [11].

### 2.11. Solubility Studies

Saturated solubility is an important parameter of nanocrystals. To determine this parameter, 1 mg of optimized KA nanocrystals was dispersed in several solvents, such as distilled water, ethanol, methanol, acetone and buffers with a pH of 7.4 and 6.8. After 1 h the sample solution was agitated at 37 °C at 100 rpm. Subsequently, Whatman filter paper having a pore size of 0.2 µm was used for filtration. An ultraviolet spectrophotometer was used to analyze each sample at 268 nm. The experiment was repeated in triplicate, and the results were averaged.

### 2.12. In Vitro Release Profile

To examine the in vitro release of the KA nanocrystals, Franz diffusion cell (IPS Lahore, Pakistan) was used. A semipermeable membrane (Tuffryn membrane) was used to separate the donor and receptor compartments. To simulate biological conditions, a buffer at a pH of 7.4 was used in the receiving chamber. Ten milligrams of the drug’s nanocrystals was loaded in the donor chamber. The temperature of the device was adjusted to 32 ± 1 °C. Aliquots were taken from the receiving chamber at intervals of 0.5, 1, 2, 4, 8, 16 and 24 h. After sampling and adjusting the tank conditions, an equal amount of fresh blank acetate buffer was added to the receiving compartment. The aliquot was filtered through a syringe filter and then analyzed with the aid of a double-beam spectrophotometer. The research was carried out three times, and the results were averaged with the SD. The experiment finished with the graphing of data on the changes in concentration over time [16].

### 2.13. Vibrational Analysis

ATR-FTIR (Perkin, Elmer, UK) analysis was performed for the nanocrystals loaded with KA and the blank preparation. A nanocrystal preparation equal to 1 mg was placed on the ATR-FTIR diamond crystal, the sample was bonded to the sample holder, and the sample was pressed with a force of 30 N. The sample was scanned to record the spectrum. This process was repeated three times for each sample, and the results were averaged.

### 2.14. Statistical Analysis

The statistical analysis of the experimental data was expressed as the average value of at least three measured values plus the SD. For the statistical analysis, the sample t-test and the one-way analysis of variance were used, and later SPSS 2021 software was used to make the comparison. The least significant level was a *p*-value of 0.05 or less.

## 3. Results and Discussion

### 3.1. Stability Studies

Stability is the ability of a preparation to retain its physical appearance, with no separation of phase or any other kind of physical change during storage [15]. The stability of KA nano-suspensions (before converting them into crystals) during centrifugation and storage was tested at different temperatures (high 40 °C and low 4 °C) for 60 days. There was no sign of phase separation in formulations F1 to F3 after storage for more than 2 months; F4 and F5 were unstable. The optimized formulations (F1-F3) were clear and uniform, without signs of separation or any other unpredictability during the preparation process and selected for further analysis [17].

### 3.2. Particle Size, Zeta Potential and DI of KA Nanocrystals

The best droplet dimension of nanocrystals is 100 to 400 nm. The PS and DI of nanocrystals have a great influence on important properties, such as drug release kinetics, drug distribution and local drug penetration. The Malvern Zetasizer Nano ZS was used to evaluate the load on the nanocrystals, their size and their DI. The PS, zeta potential and DI of the blank nanocrystal formulation, F1, and of the KA-loaded nanocrystals F2 and F3 are shown in Table 2. The formulations without KA had a droplet size of 136.5 ± 1.8 nm, zeta potential of −12.5 ± 2.3 mV, and DI of 0.44 ± 0.1. The size of the nanocrystals increased considerably by 150 ± 2.8 nm, with a high zeta potential of −15.2 ± 4.1 mV and a DI of 0.36 ± 0.4, when KA was added to the formulation (F2). In formula F3, the PS of the nanocrystals was 110 ± 3.2 nm, the zeta potential was −20.7 ± 3.5 mV and the DI was 0.48 ± 1.5. The enhancement of the size of the nanocrystals in F2 was due to the addition of KA. The reduced size of the nanocrystals in F3 was due to the increased mixing time [18]. The PS was smaller due to the long mixing time and resulted in higher stability, as shown by the zeta potential (−20.7 ± 3.5 mV) of specific nanocrystals. Due to a high zeta potential, advanced formulations displayed high stability [11]. F3 had an extremely high zeta potential (−20.7 ± 3.5 mV). Since the charges between the nanocrystals were equal, they showed greater repulsion, which favored formulation stability [9].

### 3.3. Drug Content and Entrapment Efficiency

The drug content of the KA nanocrystal preparations is shown in Table 3. About 200 mg of KA was used for the development of F2 and F3. The drug content of F2 was 82.5% and of F3 was 85%. The entrapment efficiency of F2 was 85 ± 3.4% and of F3 was 90 ± 1.9%. The prepared formulations had adequate drug content and entrapment efficiency in this research trial [14]. The high drug content showed that the drug had been precisely loaded into the formulation and had little degradation and few handling errors. Based on its stability, drug content and entrapment efficiency, F3 was considered the optimal formulation and was tested further for various characterization [17]. 

### 3.4. Differential Scanning Calorimetry

**Figure 1:**
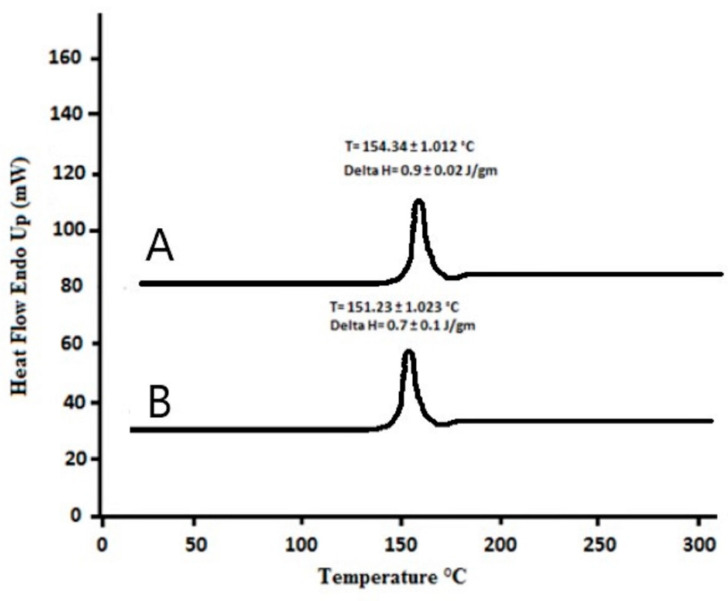
DSC thermogram of (A) pure KA and (B) KA-loaded nanocrystals (F3).

DSC thermograms determine the degree and state of the crystallinity matrix of lipid dispersions and semisolid structures and find the melting point and crystallization form of materials such as solid-lipid nanoparticles or nanocrystals [18]. KA and KA nanocrystal powder were evaluated by DSC (Figure 1). KA powder had a distinct sharp endothermic melting peak at 154.34 ± 1.012 °C in accordance with its melting point, which was an indication of the extremely crystalline character of this material. The DSC thermograms of KA nanocrystals showed that the endothermic peak was near the melting point, but the endothermic peak of KA was not observed in the freeze-dried KA nanocrystals of the physical mixture. This showed that the physical state of the KA had been transformed from a crystal-like form to an amorphous form [18]. The nanocrystals loaded with KA had an endothermic peak of KA with a slight intensity of the melting point at 151.23 ± 1.023 °C and an enthalpy of 0.7 ± 0.1 J/mol. The presence of excipients and the attenuation of the drug with several inactive materials may have lowered the melting point [12]. The melting point of the KA preparation showed that there was no interaction between the active part and the excipient in the nanocrystal preparation. The melting peak of KA with high enthalpy indicated the crystalline nature of the formulation due to the solid state [13]. 

### 3.5. Scanning Electron Microscopy

**Figure 2:**
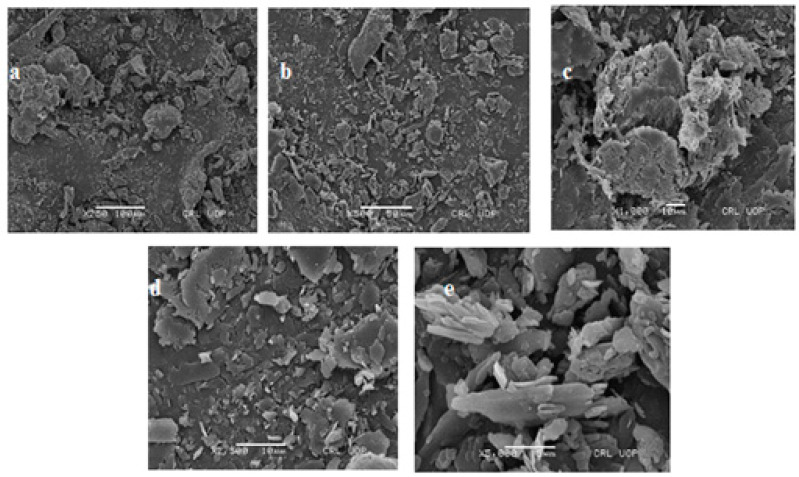
SEM images of F2 formulation of KA-loaded nanocrystals: (**a**) 250×, (**b**) 500×, (**c**) 1000×, (**d**) 2500× and (**e**) 5000×.

A high-power scanning electron microscope was used to observe the surface morphology of the nanocrystals loaded with KA. Figure 2a shows the surface morphology of KA nanocrystals at a resolution of 250×. Figure 2b to e shows the morphology at 500, 1000, 2000, 2500 and 5000 × magnification. The views confirm that the surface morphology of the KA crystal is rough, as shown in Figure 2a. Crystal particles accumulate, and KA crystals can be seen in the preparation. It is basically the KA peak, and the Carbopol peak disappears owing to the improved binding of the lipid matrix and KA (Sanna et al., 2010). The morphology of the nanocrystal surface determined the crystal size to be 120 to 140 nm (Kavoosi et al., 2013). In Figure 2e, at higher magnification, the nanocrystal surface morphology is more obvious. In the image, nanoscale crystals with a smooth surface are seen, indicating that the crystals are evenly dispersed in the preparation. The average size of KA nanocrystals was confirmed by droplet size analysis and found to be less than 200 nm, as revealed in Table 2.

### 3.6. X-ray Diffraction Studies

**Figure 3:**
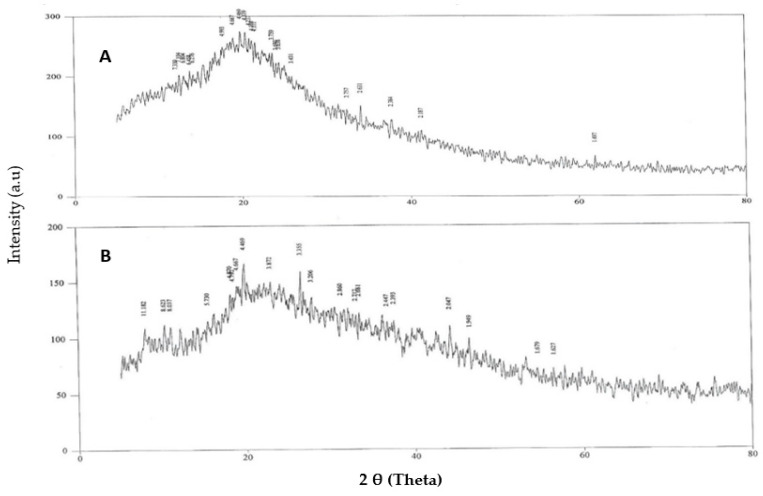
XRD thermogram of (**A**) pure KA and (**B**) KA-loaded nanocrystal formulation.

With a 2-theta of 5 to 80 degrees, the patterns of pure KA and nanocrystals loaded with KA were studied at a scanning speed of 4 degrees per minute as shown in Figure 3. The XRD spectrum of pure KA showed that the drug peaks were close to a 2-theta of 15.6857, 17.5783, 23.8135, 25.0052, 26.2619, 27.2455, 29.1017 and 42.6894; this indicated the existence of crystalline properties. The diffraction patterns of KA and Carbopol-934 were with a 2-theta of 15.8295, 20.8869, 21.3359, 30.9059, 33.6395, 34.9483, 35.4005, 42.9524 and 53.88326. Due to the uneven mixing of the drug and the excipients, the peaks of KA and nanocrystals of KA were similar. Furthermore, the Carbopol-934 layer was effectively encapsulated in the center of the nanocrystal preparation, so the KA peak disappeared [19].

### 3.7. Solubility Studies

**Figure 4:**
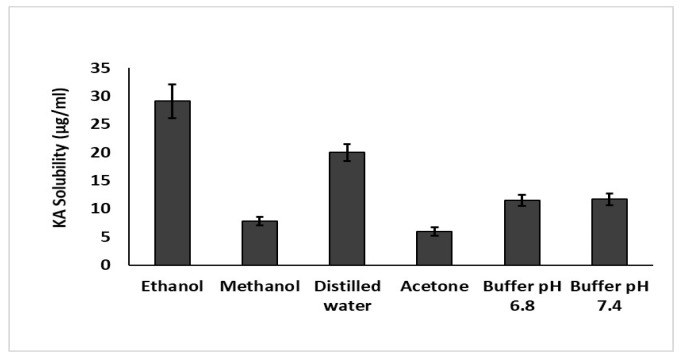
Solubility studies of KA in different solvents.

To verify the maximum solubility of the drug in the relevant solvent and to analyze the solubility curve of KA as an active moiety, distilled water, ethanol, methanol, acetone and buffers at a pH of 7.4 and 6.8 were used. It was found that KA solubility in distilled water was 20.05 ± 0.42 μg/mL; in methanol it was 7.85 ± 0.15 μg/mL, which is three times lower than in distilled water; and in acetone it was 6.02 ± 0.15 µg/mL. The lowest solubility was found in acetone and methanol due to a significant increase in the surface area of the prepared nanocrystals (*p* < 0.05), which increased the KA solubility. As reported in previous literature, the solubility of lipophilic compounds can be increased by hydrophilic inactive ingredients such as Carbopol [20]. The complete data of solubility has been shown in Figure 4.

### 3.8. In Vitro Drug Release

**Figure 5:**
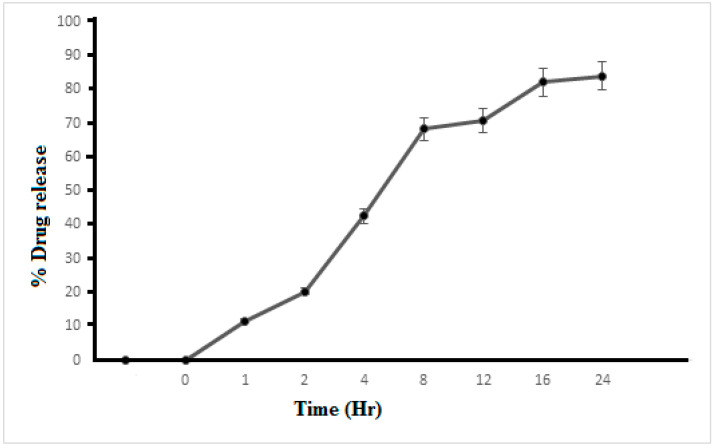
Percentage of release of KA from nanocrystals as a function of time.

Drug release from the prepared nanocrystalline formulation was measured for 24 h. The release pattern of F3 is shown in Figure 5. It was observed at 0, 1, 2, 4, 8, 12, 16 and 24 h, and the results were recorded as 0, 11.52 ± 0.21%, 20.3 ± 0.37%, 42.53 ± 0.88%, 68.26 ± 0.50%, 70.90 ± 1.10%, 82.17 ± 1.12% and 83.93 ± 1.22%, respectively. It can be seen that the release of the KA from the nanocrystalline preparation was prolonged for a period of 24 h. Releasing the drug from the formulation at a controlled rate can affect the bioavailability of the drug [19,20]. The drug had a slow and sustained release when it was encapsulated in the nanocrystal; if the drug was loaded on the outer layer of the matrix, the release was rapid and volatile [21]. The sustained release and sustained release rate of the KA nanocrystal was determined by the mechanism of the lower diffusion rate of the drug from the core of the nanocrystals and the lesser dissolution of the drug into the dissolution environment, which can be attributed to crystallization after 24 h [22]. The release data of the KA nanocrystals (F3) fit the Korsmeyer‒Peppas equation. When n is 0.350 and R^2^ is 0.9511, formulation F3 shows the Fick diffusion mechanism of KA released from nanocrystals.

### 3.9. ATR-FTIR

**Figure 6:**
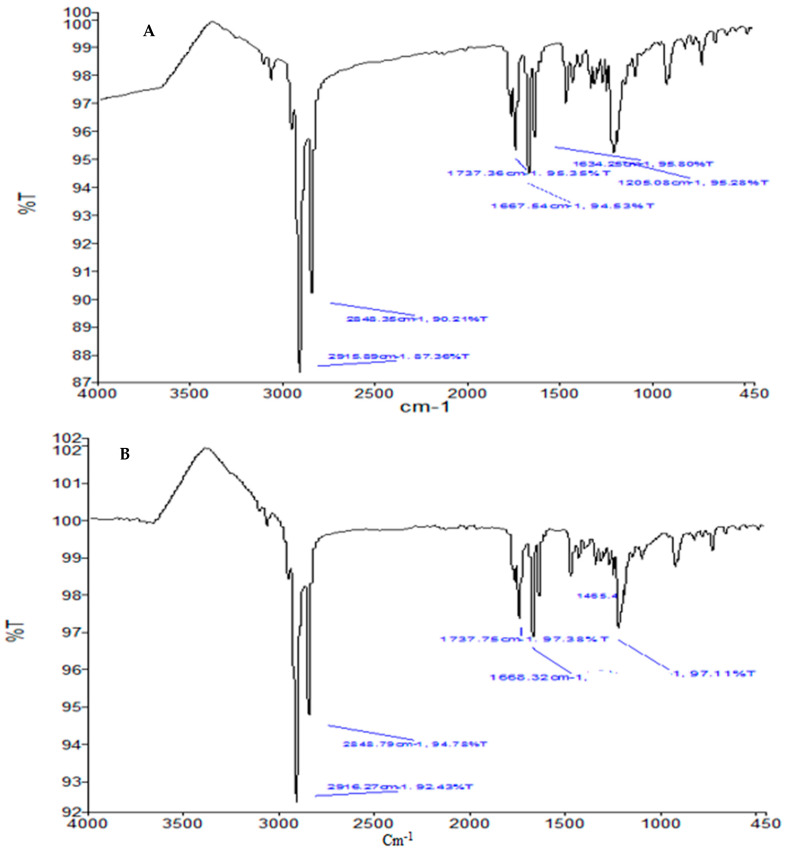
FTIR spectra of (**A**) KA and (**B**) KA-loaded nanocrystal formulation (F3).

To discover the chemical interactions between the different components of KA and KA-loaded nanocrystals, an ATR-FTIR study was conducted. ATR-FTIR spectra of the KA and KA-loaded nanocrystals with the same composition are shown in Figure 6A,B. Absorption bands were detected at 3263 cm^−1^ and 3144 cm^−1^ due to the stretching vibration of the O-H group. Due to C-H stretching, the bands appeared at 2925 cm^−1^ and 2833 cm^−1^, and C-O extension caused the bands to appear at 1069 cm^−1^. The carbonyl stretch appears at 1696.5 cm^−1^, and the C-C stretch appears at 1450.2 cm^−1^. No characteristic KA peak is seen in the spectrum of the FTIR formula. The absence of characteristic drug peaks is the main indicator of successful drug insertion into the core of nanocrystalline preparations [17]. No changes in the characteristic peaks related to the C-O and C-C groups of KA were detected by ATR-FTIR on the basis of the physical mixture of KA, i.e., KA-loaded nanocrystals. Therefore, no chemical interaction between the drug and the excipients was noted.

## 4. Conclusions

The KA nanocrystals were effectively fabricated using high-pressure homogenization to improve the water solubility of the drug. KA nanocrystals had improved solubility at both an acidic pH and a basic pH. The improvement in the solubility of KA may potentially provide opportunities for the development of cost-effective dosage forms that will produce similar or improved pharmacological effects but at a lower dose and frequency as compared with products already available. All the characterizations indicate that KA nanocrystals have been successfully engineered as a promising carrier system for successfully transporting drugs that are lipophilic.

## Figures and Tables

**Table 1:** Various compositions of KA nanocrystals.

Formulation Code	Carbopol-934 (*w*/*w*)	Tween-80 (*w*/*w*)	Kojic Acid (*w*/*w*)	Water (*w*/*w*)
**F1**	0.1	0.5		49.4
**F2**	0.5	0.5	50	49.0
**F3**	1.0	0.5	50	48.5
**F4**	1.5	0.5	50	48.0
**F5**	2.0	0.5	50	47.5

**Table 2:** Particle size, zeta potential and DI of KA-loaded nanocrystals.

Formulations	Particle Size (nm)	Zeta Potential (mV)	DI
**F1**	136.5 ± 1.8	‒12.5 ± 2.3	0.44 ± 0.1
**F2**	150 ± 2.8	‒15.2 ± 4.1	0.36 ± 0.4
**F3**	110 ± 3.2	‒20.7 ± 3.5	0.41 ± 1.5

**Table 3:** KA content and encapsulation efficiency.

Formulation	Kojic Acid Concentration (mg)	Kojic Acid Obtained (mg)	Kojic Acid Content (%)	Percentage Entrapment Efficiency ± SD
F2	200	165	82.5	82.5 ± 3.4
F3	200	170	85.0	85.0 ± 1.9

## Data Availability

Data will make available on request.
